# Factors contributing to men’s grief following pregnancy loss and neonatal death: further development of an emerging model in an Australian sample

**DOI:** 10.1186/s12884-020-03514-6

**Published:** 2021-01-07

**Authors:** Kate Louise Obst, Melissa Oxlad, Clemence Due, Philippa Middleton

**Affiliations:** 1 School of Psychology, University of Adelaide, Adelaide, South Australia; 2 South Australian Health and Medical Research Institute, Adelaide, South Australia

**Keywords:** Pregnancy loss, Neonatal death, Miscarriage, Stillbirth, Termination of pregnancy, Foetal anomaly, Men, Grief

## Abstract

**Background:**

Historically, men’s experiences of grief following pregnancy loss and neonatal death have been under-explored in comparison to women. However, investigating men’s perspectives is important, given potential gendered differences concerning grief styles, help-seeking and service access. Few studies have comprehensively examined the various individual, interpersonal, community and system/policy-level factors which may contribute to the intensity of grief in bereaved parents, particularly for men.

**Methods:**

Men (*N* = 228) aged at least 18 years whose partner had experienced an ectopic pregnancy, miscarriage, stillbirth, termination of pregnancy for foetal anomaly, or neonatal death within the last 20 years responded to an online survey exploring their experiences of grief. Multiple linear regression analyses were used to examine the factors associated with men’s grief intensity and style.

**Results:**

Men experienced significant grief across all loss types, with the average score sitting above the minimum cut-off considered to be a high degree of grief. Men’s total grief scores were associated with loss history, marital satisfaction, availability of social support, acknowledgement of their grief from family/friends, time spent bonding with the baby during pregnancy, and feeling as though their role of ‘supporter’ conflicted with their ability to process grief. Factors contributing to grief also differed depending on grief style. Intuitive (emotion-focused) grief was associated with support received from healthcare professionals. Instrumental (activity-focused) grief was associated with time and quality of attachment to the baby during pregnancy, availability of social support, acknowledgement of men’s grief from their female partner, supporter role interfering with their grief, and tendencies toward self-reliance.

**Conclusions:**

Following pregnancy loss and neonatal death, men can experience high levels of grief, requiring acknowledgement and validation from all healthcare professionals, family/friends, community networks and workplaces. Addressing male-specific needs, such as balancing a desire to both support and be supported, requires tailored information and support. Strategies to support men should consider grief styles and draw upon father-inclusive practice recommendations. Further research is required to explore the underlying causal mechanisms of associations found.

**Supplementary Information:**

The online version contains supplementary material available at 10.1186/s12884-020-03514-6.

## Background

Despite continued global advancements in reproductive healthcare, both pregnancy loss and the death of a newborn baby within the first 28 days following birth (neonatal death) continue to be devastating realities for many families. The pervasive psychological and emotional impacts of parents’ grief following pregnancy loss and neonatal death are now well-recognised [1–4]. Parents frequently report experiences of stigma, shame and disenfranchisement through minimisation of their loss from others, which can complicate their grief [5–9]. Men’s experiences of pregnancy loss and neonatal death have been under-explored in comparison to women. However, a growing body of research has highlighted the importance of investigating men’s perspectives, given potential gendered differences concerning grief, help-seeking and service access [10–17]. For example, quantitative studies comparing heterosexual couples’ experiences following pregnancy loss and neonatal death suggest that men typically experience less intense and enduring levels of grief than women [18–23]. However, a smaller number of studies have found similar grief intensity between men and women [24, 25], or even higher levels of grief in men [26]. Broader research on grief also demonstrates potential differences in grief styles for men and women, with a general classification made between instrumental (action-focused coping) and intuitive (emotion-focused coping) styles [27]. Following pregnancy loss and neonatal death, studies suggest that men may engage in more instrumental grieving styles, which includes using activities, distraction or problem-solving approaches to grief, as opposed to intuitive styles which use emotion-focused approaches including outward displays of crying, talking about grief, or seeking social support [9, 16, 28–35].

**Fig. 1:**
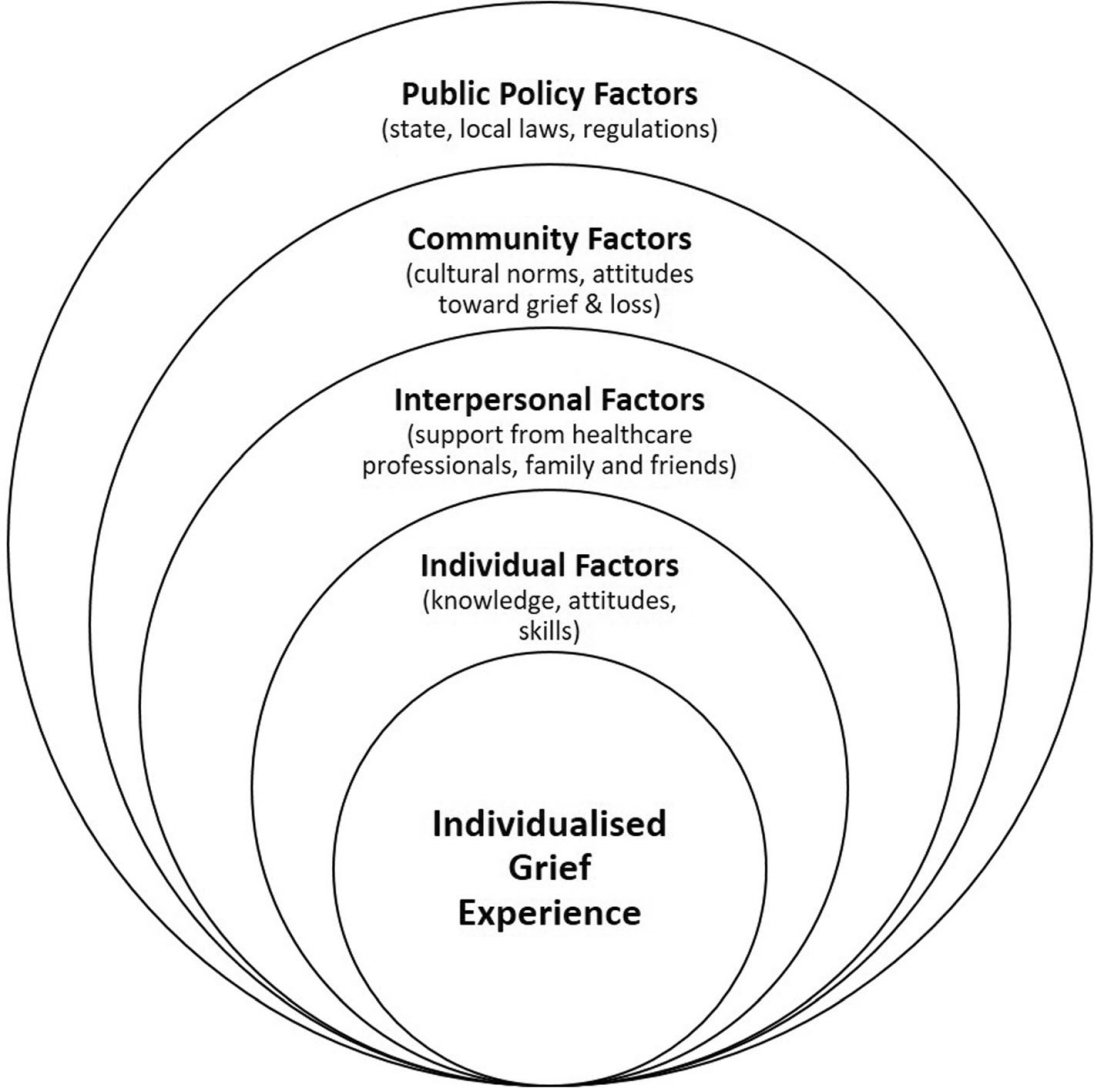
Socioecological model of men’s grief. Socioecological model of men’s grief, demonstrating the factors contributing to men’s grief following pregnancy loss and neonatal death at the individual, interpersonal, community and public policy levels. This image was generated by the authors for a previous publication [36]

Our recent systematic review of men’s grief following pregnancy loss and neonatal death emphasised the importance of examining grief from a holistic, socioecological perspective to understand the varied factors which can contribute to men’s experiences (see Fig. 1) [36]. At the individual level, factors contributing to men’s grief include demographic elements (e.g., age, religion, ethnicity), pregnancy loss/neonatal death history and number of living children. Regardless of gestational/newborn age of the baby, previous research also suggests that attachment is a particularly strong predictor of men’s grief intensity. Although early quantitative research measured ‘attachment’ using increasing gestational age or whether or not men viewed an ultrasound of their developing baby [20, 22, 23, 37, 38], qualitative studies have suggested that a broader exploration of prenatal attachment (e.g., through everyday interactions with the developing baby) may be more important in determining the intensity of men’s grief response [10, 12, 13, 16, 30, 32, 34].

At the interpersonal level, men’s interactions with others have been found to have implications for shaping their grief experience. Qualitative studies have pointed to the importance of whether men felt acknowledged as a grieving father from family, friends and healthcare professionals; where there was a lack of recognition for men as grieving fathers, grief intensity worsened [9, 10, 12, 33, 39]. Throughout the qualitative literature, heterosexual men’s role primarily as a ‘supporter’ to their female partner has remained a consistent and dominant theme. This role has often been reported as hindering men’s expressions and experiences of grief [10, 12, 13, 16, 33, 34, 40]. However, a smaller number of studies have also suggested potential benefits of this role, particularly among people who are instrumental grievers, for whom this role could provide purpose [30, 41].

At the broader community level, qualitative studies have also consistently noted that men’s experiences are shaped by social attitudes concerning the legitimacy of parents’ grief, as well as gendered expectations surrounding how (or if) men should openly display emotion [9, 10, 12, 16, 29]. These expectations were related to masculinity ideals, which often prescribed being strong or stoic in the face of loss [12, 13, 16, 32, 33].

Finally, at the system/policy level, experiences within the healthcare system following pregnancy loss or neonatal death have been established as fundamental to shaping bereaved parents’ grief experience [4, 9, 42]. For some men specifically, the context of woman-centred care in hospital (when applicable to the type of loss) has been found to be isolating and can worsen grief outcomes [10, 12, 34]. Also in relation to systems issues, research indicates that policies regarding bereavement leave within workplaces typically differ for men and women, with some men reporting less access to paid leave following their loss than women [10, 29, 33]. As many men have reported returning to work soon after pregnancy loss or neonatal death, bereavement leave policies may play a role in grief outcomes [36].

In addition to the research on factors relating to men’s grief, several studies have explored various factors relating to grief intensity in women following pregnancy loss and neonatal death [43–50] and couples [23, 38, 51–53]. Most of these studies have examined individual, interpersonal, community or system-related factors separately, rather than together in a single model. Similarly, with the exception of Riggs et al. [17] who explored relationships between grief, psychological distress, stigma, help-seeking and social support, the studies outlined above concerning factors related to grief for men have also focused on specific variables such as the duration of pregnancy or viewing an ultrasound image. Importantly, no previous research has considered factors relating to different styles of grief, which may be important, given that studies have suggested gendered grieving styles [27]. Using the socioecological model of men’s grief developed in our previous systematic review as a basis [36], this study aimed to quantify and further explore the factors which contribute to men’s grief, with a particular focus on previously under-explored determinants. Specifically, we sought to determine the factors associated with grief intensity following pregnancy loss and neonatal death, as well as the factors associated with intuitive and instrumental grief styles.

## Methods

### Participants

Ethical approval for the study was granted by the University of Adelaide Human Research Ethics Committee on the 5th of June, 2019 (approval code HREC-2018-273). Participants were Australian men who had experienced the loss of a baby at any stage of gestation to miscarriage, ectopic pregnancy, medical termination of pregnancy for nonviable foetal anomaly (TOPFA), stillbirth or neonatal death. Inclusion criteria were that participants were aged 18 years of age or older and had experienced pregnancy loss or neonatal death in Australia within the last 20 years. Although potentially open to recall bias, this timeframe was selected to maximise the potential pool of eligible respondents. Of 277 participants who commenced the survey, 228 completed all items and were included in the final sample reported here (completion rate = 82%). There were no apparent differences between completers and non-completers on demographic characteristics. At the time of survey completion, participants were aged between 19 and 60 years (*M* = 36, *SD* = 7.4). At the time of loss, they were aged between 18 and 58 years (*M* = 32, *SD* = 5.5*)*. See Table 1 for a summary of participant characteristics at the time of survey completion.

**Table 1:** Participant characteristics

	Category	***N*** (%)
Ethnicity	Australian	194 (85%)
	Other^a^	34 (15%)
Sexual orientation	Heterosexual	224 (98%)
	Bisexual	3 (1.5%)
	Homosexual	0 (0%)
	Transgender	0 (0%)
	Rather not answer	1 (0.5%)
Highest level of education	High School	54 (24%)
	Technical and Further Education (TAFE)/Trade	83 (36%)
	Undergraduate Degree	58 (25%)
	Postgraduate Degree	33 (15%)
Marital status	Married	186 (82%)
	In a relationship	35 (15%)
	Divorced	1 (0.5%)
	Separated	4 (1%)
	Never married/single	2 (2%)
Area of residence^b^	Major city	131 (58%)
	Inner regional	64 (28%)
	Outer regional	28 (12%)
	Remote/very remote	4 (2%)
Number of losses	One	138 (61%)
	Two–three	15 (7%)
	Four–five	47 (21%)
	Six or more	28 (12%)
Loss type reflected on for the survey	Ectopic pregnancy	5 (2%)
	Termination of pregnancy for foetal anomaly (TOPFA)	30 (13%)
	Miscarriage	69 (30%)
	Stillbirth	77 (34%)
	Neonatal death	47 (21%)
Time since loss	Less than one year	65 (28%)
	1–2 years	40 (18%)
	3–5 years	59 (26%)
	6–10 years	43 (19%)
	11–15 years	10 (4%)
	16–20 years	11 (5%)

^a^Other ethnicities reported by participants include: European (8%), Asian (2%) and New Zealander (2%)

^b^Based on Australian Bureau of Statistics classification of remoteness

### Procedure

A web-based survey was developed by the authors (see Additional file 1), hosted by the online platform *SurveyMonkey*. This survey was developed for the purposes of the current study, and has not been published elsewhere. Extensive consultation and piloting was undertaken with members of a reference group (including Australian fathers and mothers who had experienced pregnancy loss/neonatal death, grief counsellors and pregnancy loss/neonatal death support workers and researchers) as part of the broader program of research to form the final survey. Initially, preliminary discussions were held with individual members of the reference group concerning the types of measures used and questions to be asked, in line with the socioecological model of men’s grief. With this feedback, the first author (KO) drafted a full survey. In the two successive rounds of piloting, members of the reference group reviewed updated drafts of the survey in full and were invited to provide suggestions for revision. Although major concepts remained the same, the ordering, inclusion and wording of questions and final measures selected, were edited and refined according to feedback to ensure both sensitivity and ease of understanding.

Potential participants were invited to take part in the survey via advertisements through Australian pregnancy loss and neonatal death support and advocacy organisations. These included Pillars of Strength, Bears of Hope, Sands Australia, Still Aware, Miracle Babies Foundation, SIDS and Kids SA, and the Australian Perinatal Loss Centre. Following ethics approval, these organisations were contacted by the first author via email or telephone to discuss the study. All organisations agreed to share a study flyer and information through either social media platforms (primarily Facebook), newsletters, and/or organisation websites.

The study flyer contained brief information about the survey and the online survey link, which opened to a covering page with a preamble providing potential participants with detailed information about the study. After reading the study preamble, participants provided passive consent, a method of consent approved by the University of Adelaide Human Research Ethics Committee, by choosing to commence the survey and submit their responses. In recognition of the sensitivity of the topic and potential for participants to experience emotional distress in reflecting on their experience of loss, a comprehensive distress protocol was developed and articulated to participants. This included providing contact details for national pregnancy loss telephone support lines at the beginning and end of the survey. No concerns regarding participant distress were raised during the research.

The survey took approximately 30 min to complete. Depending on participant responses, skip logic was incorporated to hide questions which were irrelevant to individual experiences, often resulting in a shorter completion time (*M = *22 mins). The number of items/questions presented to participants who completed they survey therefore ranged between 110 and 130. Participation in the survey was voluntary and anonymous. Data collection occurred between June and August 2019. Data were exported from the online *SurveyMonkey* platform and stored on a secure university-approved network at the University of Adelaide.

### Measures

Participants completed questions relating to demographic characteristics (age, ethnicity, education, occupation, sexual orientation, marital status, religion and postcode), along with questions about their pregnancy and loss history. Definitions for the death of a baby during pregnancy or shortly following birth vary, with gestational cut-offs for classification differing between countries. In Australia, a miscarriage is defined as the death of a baby in-utero before 20 weeks’ gestation and occurs for approximately 20% of pregnancies [54]. In 1–2% of pregnancies, an ectopic pregnancy occurs when the fertilised ovum implants outside of the uterus, most commonly in the fallopian tube [54–56]. A stillbirth is defined as the loss of a baby from at least 20 weeks’ gestation or over 400 g in weight, occurring for 7.1 per 1000 births [57]. Neonatal death refers to the death of a newborn infant within the first 28 days of life and occurs for 2.5 per 1000 live births [57]. A congenital anomaly is diagnosed in approximately one in 22 pregnancies in Australia [58]. National data on TOPFA is not collected in Australia. However, it has been estimated that most parents whose pregnancies are deemed life-limiting or affected by chromosomal anomalies elect to medically terminate; usually before, or soon after, 20 weeks of gestation [59]. Where more than one type of pregnancy loss or neonatal death had been experienced, participants were asked to reflect on only one type of their choice for the remainder of the survey. An option to comment on other losses was provided at the end of the survey. Participants then completed a mix of questions developed by the authors as well as standardised measures.

In line with the literature on the ‘supporter role’ relating to men’s grief [10, 12, 13, 16, 36, 40, 60], two author-developed measures were included to determine the extent to which men perceived this to be their role, and whether they felt it interfered with their grief. Participants responded to these questions on a five-point Likert scale from 1 = *strongly disagree* to 5 = *strongly agree*. Higher scores indicated that men perceived their role to be a supporter to their female partner and family after the loss and that their supporter role had a larger impact on their ability to grieve. Scales were also developed to determine the extent to which participants felt their grief was recognised by others, namely: their partner, family, friends, health professionals, and the wider community. For these, participants responded on a five-point Likert scale from 1= *not at all* to 5 = *extremely*. Higher scores were indicative of higher levels of recognition for their grief. Participants were also asked a series of questions about their experiences of returning to work, including whether they were offered leave, and what type of leave they were offered (detailed results  published elsewhere; see [103]). If they had contact with a hospital as part of their loss experience, they were also asked about the extent to which they felt included (from 1 = *not at all* to 5 = *extremely*; where higher scores indicated a greater sense of inclusion), and whether they were offered information on grief for fathers (yes/no). The six included standardised measures are outlined below.

#### Paternal Antenatal Attachment Scale (PAAS)

A modified version of the Maternal Antenatal Attachment Scale (MAAS), the PAAS assesses both the quality and strength of the subjective experience of the father’s attachment to the developing baby [61, 62]. Comprising 16 items forming two subscales (*Quality of Attachment* and *Time in Attachment*), the PAAS is answered using five-point Likert scales, where higher scores indicate stronger attachment to the baby. Although only a small number of previous studies have used the PAAS, relationships have been found with related measures including relationship quality, mental health, increasing gestational age, and father identity [63, 64]. Previous research also supports the reliability and validity of the PAAS, with reports of high internal consistency (Cronbach’s alpha = 0.83) [62]. For this study, the final question of the scale “If the pregnancy was lost at this time (due to miscarriage or other accidental event) I expect I would feel …” was omitted, given that participants had experienced a pregnancy loss or neonatal death. Internal consistency of this 15-item version in this study was also high (Cronbach’s alpha = 0.83).

#### Perinatal Grief Scale-33 (PGS-33)

Designed to quantify bereaved parents’ grief based on emotional responses, the PGS-33 assesses thoughts and feelings associated with perinatal loss [65]. The overall scale comprises three subscales: *Active Grief* (outward expressions of grief including crying, sadness and missing the baby), *Difficulty Coping* (difficulties with daily activities and relating to others) and *Despair* (feelings of hopelessness and worthlessness). Participants rate each item on a five-point Likert scale ranging from 1 = *strongly agree* to 5 = *strongly disagree*, with higher scores indicating more intense grief. The PGS-33 is the most common grief scale used among the perinatal loss literature and has been extensively evaluated, with psychometrically sound properties reported (including Cronbach’s alphas between 0.92 and 0.96) [66, 67]. Internal consistency for the full measure was also high in this study (Cronbach’s alpha = 0.94). Although questions remain surrounding the accuracy of using the PGS among men, as it may not be sensitive to instrumental grieving styles [36], given a current lack of alternative grief measures specifically for men following pregnancy loss/neonatal death, we decided that in conjunction with the Grief Patterns Inventory (described below), this was the best available measure to adopt.

#### Grief Patterns Inventory-Revised (GPI-10)

A measure developed to assess an individuals’ general grieving pattern, the GPI indicates a tendency toward either an instrumental or intuitive grieving style. The original measure comprised 24 items containing true-false responses; however, a revised version containing ten items (five items each for the instrumental and intuitive styles) was used in the current study to reduce respondent burden [68, 69]. A pilot study of the 10-item version reported moderate inter-correlations between subscale items, along with a significant negative correlation between the intuitive and instrumental subscales (*r* = −.525) [68]. Although alpha coefficients were not reported for the 10-item version, research demonstrates acceptable internal consistency for the original version (Cronbach’s alphas ranging between 0.71 and 0.76) [70]. In this study, a similar level of internal consistency was found (Cronbach’s alpha = 0.71). Items are rated on a five-point Likert scale from 1 = *strongly disagree* to 5 = *strongly agree,* with instrumental items reverse-scored. As such, potential total scores ranged from 10 to 50, with lower scores indicating a more instrumental style, and higher scores indicating a more intuitive style. As applied previously [69], categorisation of grief styles was made as follows: 10–23 = instrumental; 24–36 = blended; 37–50 = intuitive.

#### Crisis Support Scale (CSS)

The CSS is a measure of social support received from family and friends following a traumatic event (in this case, pregnancy loss or neonatal death). Comprising seven items relating to the availability of others, emotional support, and practical help, respondents rate their agreement to the items on a seven-point Likert scale, ranging from 1 = *never* to 7 = *always*. In the original scale, participants responded to two time points: just following the event (T1) and the present time (T2). However, for this study, participants were only asked to provide responses for the support that was available to them most of the time following their loss. Higher scores indicate higher levels of social support. Validation studies indicate robust psychometric properties for the scale across a range of trauma populations, including bereaved parents of infants (with Cronbach’s alphas ranging between 0.67 and 0.82; in this study, Cronbach’s alpha was 0.69) [71].

#### Conformity to Masculine Norms Inventory (CMNI)

Developed based on Mahalik’s model of gender role conformity, the CMNI assesses the extent to which an individual male does or does not conform to the actions, thoughts, and feelings reflected by broad masculinity norms [72, 73]. The original scale consists of 144 items forming 11 distinct factors. However, to reduce participant response burden, only one subscale comprising five items from the overall measure was included for this study, to determine respondents’ tendencies toward *Self-Reliance*. This subscale was chosen in line with previous literature which suggests men often feel the need to hide their grief from others, preferring to cope in isolation [10, 12, 13, 16, 29, 33]. The questions included: “I never ask for help”, and “It bothers me when I have to ask for help”. Respondents rated the degree to which they agreed with these statements on a four-point Likert scale from 1 = *strongly disagree* to 4 = *strongly agree*, with higher scores indicating a stronger tendency toward being self-reliant. Widely used in the literature, many studies have reported construct validity for the CMNI, along with discriminant validity between its subscales and high internal consistencies (Cronbach’s alpha of 0.85 for the *Self-Reliance* subscale; in this study, Cronbach’s alpha was 0.86) [73].

#### Male Role Norms Inventory-Short Form (MRNI-SF)

A measure of masculinity ideology developed by Levant et al. [74], the original MRNI comprised 57 items with seven subscales. In 2011, a 39-item revised form was proposed, followed by a 21-item short-form in 2013. For this study, the *Toughness* subscale from the MRNI-SF was used, as items closely aligned with the recurrent theme of needing to be ‘strong’ or ‘tough’ reported by men following pregnancy loss in previous literature [10, 13, 16, 32, 33]. The subscale comprises three items, including: “When the going gets tough, men should get tough”. Responses are given on a seven-point Likert scale from 1 = *strongly disagree* to 7 = *strongly agree.* Higher scores indicate higher levels of endorsement toward traditional masculine ideology [75]. Research has demonstrated sound psychometric properties for the MRNI-SF, including subscale alphas ranging from 0.79 to 0.90 [75]. In this study, Cronbach’s alpha for the 3-item *Toughness* subscale was 0.61.

### Data analysis

Analyses were performed using SPSS Statistics (V.25). Data were summarised using descriptive statistics and relationships between the variables were assessed using generalized linear modelling with a multiple stepwise approach, including a backward elimination method outlined by Sainani [76]. The generalised linear model is a flexible form of usual linear regression used to compare the effect of several variables which may have error distributions other than a normal distribution on a continuous outcome variable. Using a link function to relate the response variable to the linear model, it provides a maximum likelihood estimation of the model parameters rather than assuming a linear-response model [77]. As recommended for multivariable modelling [78], a priori selection of variables for this study was guided by the socioecological model of men’s grief identified in our previous systematic review [36]. Given the nested form of the socioecological model, variables were entered into the regression models in four (stepwise) stages. Assumptions required for generalised linear modelling were assessed prior to analysis; all necessary assumptions were satisfied. Individual-level variables were entered first, and a backward elimination process was carried out until all variables were statistically significant at the 0.5 level as recommended by Harrell [78]. This process was repeated with each of the interpersonal, community and policy/system-level variables until all had been entered into the model (see Table 2 for the variables entered at each level).

**Table 2:** Variables entered into the multiple linear regression analyses

Stage entered into the model	Variables
Stage 1 (individual variables)	Loss type; Grief style^a^; PAAS *Time in Attachment*; PASS *Quality of Attachment*; Whether men attended obstetric appointments; Whether men viewed an ultrasound image of their baby; Age at time of loss; Ethnicity; Importance of religion; Number of previous losses; Number of surviving children at time of loss
Stage 2 (interpersonal variables)	Marital satisfaction; Extent of agreement to the statement: “My role following the loss was to support my partner and family”; Extent of agreement to the statement: “I was unable to grieve, because I was too busy supporting everyone else”; Total CSS score; Extent of acknowledgement from partner; Extent of acknowledgement from family; Extent of acknowledgement from friends
Stage 3 (community variables)	Extent of acknowledgement from community; CMNI *Self-Reliance* subscale total score; MRNI *Toughness* subscale total score
Stage 4 (policy/system variables)	Extent of acknowledgement from healthcare professionals; Degree to which participants felt included in the hospital^b^; Whether employment leave was offered to men; Whether other psychosocial supports were offered to men

Note: ^a^Entered only into model 1 (dependent variable = PGS total score); ^b^Classified into a high/low level of inclusion based on original Likert scale responses (scores 1–3 = low level of inclusion and 4–5 = high level of inclusion)

While we acknowledge the debates surrounding the use of *p*-values in making decisions regarding variable selection [79, 80], the cut-off for inclusion of 0.5 (rather than the traditional choice of *a* = 0.05) used for variable selection in this study is considered to be a reasonable and conservative estimate for a multivariable model [78]. Also, to reduce the risk of bias from sparse data, backward elimination is recommended to achieve a suitable number of degrees of freedom for the model given the number of observations in the study; a general rule is that degrees of freedom should be no more than the number of observations divided by ten to reduce the risk of bias [78]. Without the use of backward elimination, our models would have violated this rule. Ultimately, our approach resulted in a suitable number of degrees of freedom for each model in this study.

#### Statistical power

There are no consistent rules for sample size requirements in linear regression [81]. However, various general recommendations have been made about minimum sample size, or sample size depending on the number of independent variables included in the model. While one general rule recommends a minimum of 100 participants regardless of the number of independent variables [82], others suggest 50 plus the number of independent variables [83], or at least 100 for less than three independent variables or 300–400 for nine or 10 independent variables [84]. Tabachnick and Fidell also suggested a sample size of 50 + 8*k*, where *k* is equal to the number of independent variables [85]. Employing recommendations to consider a minimum sample size of 50 plus the number of independent variables [83, 85], with a sample size of 228 and the number of independent variables included in the models at any one stage not exceeding 16, the current study had sufficient statistical power.

## Results

### Descriptive statistics

Descriptive statistics for continuous variables are presented in Table 3. Overall, grief scores were high, with the average score sitting above the minimum cut-off considered to be a high degree of grief according to population norms (cut-off = 91) [67]. In particular, the highest mean grief scores occurred on the *Active Grief* subscale (indicating feelings of sadness and missing the baby), and the lowest scores occurred on the *Despair* subscale (indicating feelings of worthlessness and hopelessness). On average, men experienced the lowest average grief following early losses (<20 weeks’ gestation); however, the standard deviation (*SD*) was high and the mean score still represented a high degree of grief. The average grief score following late loss (≥20 weeks’ gestation) was the highest, followed by neonatal death. Again, however, the ranges were wide and *SD*s were high, indicating substantial variation in scores. Although the mean grief score was slightly lower for losses which occurred more than 10 years ago, there was a negligible association between time since loss and total grief scores (F(2,215) = .556, *p* = .574).

**Table 3:** Descriptive statistics for continuous variables

	***N***	Mean	Range	***SD***
**Total PGS score according to loss type**				
Early loss (<20 weeks’ gestation)^a^	82	93.4	47–152	23.2
Late loss (≥20 weeks’ gestation)^b^	90	109.7	65–158	19.8
Neonatal death	46	107.0	65–151	23.4
**Total PGS score according to time since loss**				
Last 5 years	156	103.2	49–158	23.3
6–10 years ago	42	104.8	47–151	23.6
11–20 years ago	20	98.2	65–141	21.1
**Individual-level variables**				
Age at loss (in years)	225	32	17–58	5.5
Time since loss (in years)	228	4.3	0–20	55
PGS total score	218	103	47–158	23.0
Active Grief subscale	222	42.8	22–55	6.3
Difficulty Coping subscale	226	32.7	12–53	9.8
Despair subscale	226	27.2	11–54	9.1
GPI – Intuitive	225	19.6	8–24	3.8
GPI – Instrumental	227	17.6	5–25	4.1
PAAS total score	224	58.5	35–72	8.0
PAAS Quality of Attachment subscale	224	30.1	15–35	3.6
PAAS Time in Attachment subscale	228	19.7	5–28	4.5
**Interpersonal-level variables**				
Marital satisfaction at time of loss	228	4.8	1–5	0.6
Acknowledgement from partner	228	3.9	1–5	1.0
Acknowledgement from friends	228	3.0	1–5	1.1
Acknowledgement from family	228	3.4	1–5	1.2
CSS total score	226	30.9	10–48	8.3
Extent of agreement to: “My role following the loss was to support my partner and family”	228	4.5	1–5	0.8
Extent of agreement to: “I was unable to grieve, because I was too busy supporting everyone else”	228	3.3	1–5	1.3
**Community-level variables**				
Acknowledgement from community	228	2.1	1–5	1.1
CMNI Self-Reliance subscale	227	13.1	5–20	3.1
MRNI Toughness subscale	228	11.7	3–21	3.7
**Policy/system-level variables**				
Perceived extent of inclusion in the hospital	189	3.6	1–5	1.3
Acknowledgement from healthcare professionals	228	2.7	1–5	1.3

^a^Includes ectopic pregnancy, miscarriage and TOPFA at less than 20 weeks’ gestation

^b^Includes TOPFA and stillbirth at or over 20 weeks’ gestation

According to the GPI, average scores were significantly higher for the intuitive grief items compared to the instrumental grief items (*t* (223) = 4.611, *p* <.001). Men’s total reported attachment to their baby was also generally high. Specifically, scores on the *Quality of Attachment* subscale were also significantly higher than those on the *Time in Attachment* subscale (*t* (223) = 38.9, *p* <.001).

Men felt the most acknowledgement for their grief from their partners, and the least acknowledgement from the wider community and healthcare professionals. Average agreement concerning the extent to which men felt they had a supporter role following the loss was high. However, agreement regarding the extent to which this role impacted men’s ability to grieve was in the mid-range.

### Multiple linear regression models

Multiple stepwise linear regression analyses were performed to determine which variables were associated with total grief (PGS total score), intuitive grief, and instrumental grief (GPI scores). Results for the three resulting models are presented below.

#### Model 1: Total grief (PGS)

Fourteen variables were below the 0.5 significance cut-off for inclusion in the final model for men’s total grief scores, and seven of these had confidence intervals which did not contain zero (see Table 4). When adjusting for all other factors, men who lost a baby to miscarriage had a mean total PGS score of 16.5 points less than men who experienced a neonatal death. However, the confidence interval was wide, indicating a low level of precision in this estimate; this may be due to large variability in grief scores across loss types. Narrow confidence intervals, indicating higher levels of certainty, were observed for history of loss, time in attachment and overall support. Specifically, a higher number of previous pregnancy losses/neonatal deaths were associated with higher levels of grief, as were lower levels of overall support and increased time in attachment. Higher grief scores were also associated with lower levels of acknowledgement of grief from friends, as well as higher levels of agreement to the statement: “I was unable to grieve, because I was too busy supporting everyone else”. However, the opposite was observed for acknowledgement from family, with men experiencing higher levels of grief with more acknowledgement. Again, though, confidence intervals were wide for these factors, indicating less certainty in the precision of the estimates.

**Table 4:** Multiple stepwise linear regression for PGS total score (*n* = 204)

	*B*	95% CI	SE *B*	*β*	*p*
**Variables**					
***Loss focus***					
Ectopic pregnancy	−4.09	(−18.78–10.60)	7.49	−.02	.59
TOPFA	−6.41	(−14.08–1.26)	3.91	−.09	.10
Miscarriage	**−16.48**	**(−23.01 – −9.95)**	3.33	−.32	<.001
Stillbirth	1.42	(−4.57–7.42)	3.06	.03	.64
Neonatal death	Ref	–	–	–	–
PAAS Time in Attachment subscale	**2.10**	**(1.60–2.61)**	.26	.44	<.001
Age at loss (in years)	0.39	(−0.05–8.37)	.23	.09	.09
Number of previous losses experienced	**1.84**	**(0.18–3.49)**	.85	.10	.03
Marital satisfaction at the time of loss	−3.60	(−7.01–0.19)	1.74	−.10	.04
Agreement to the statement: My role following the loss was to support my partner and family”	−2.48	(−5.45–0.49)	1.52	−.09	.10
Agreement to the statement: “I was unable to grieve, because I was too busy supporting everyone else”	**3.45**	**(1.45–5.45)**	1.02	.21	<.01
CSS total score	**−.69**	**(−1.12 – − 0.26)**	1.02	−.24	<.01
Extent of acknowledgement of grief from family	**3.39**	**(0.74–6.05)**	1.36	.16	<.01
Extent of acknowledgement of grief from friends	**− 2.89**	**(−5.75 – − 0.03)**	1.46	−.13	.05
Extent of acknowledgement from wider community	− 1.92	(−4.44–0.59)	1.28	−.11	.13
CMNI *Self-Reliance*	.47	(− 0.32–1.25)	.40	.07	.25
***Workplace leave***					
Employment leave offered	21.44	(−11.11–53.99)	16.61	.47	.19
Employment leave not offered	21.12	(−11.61–53.86)	16.70	.39	.21
Did not inform employer of loss	Ref	–	–	–	–
***Other workplace supports***					
Other supports offered	−23.42	(−56.45–9.60)	16.85	−.49	.17
Other supports not offered	−25.75	(−59.17–7.66)	17.05	−.58	.13
Did not inform employer of loss	Ref	–	–		–

#### Model 2: Intuitive grief (GPI)

Thirteen variables met the 0.5 significance cut-off for inclusion in the final model for intuitive grief, although only one had a confidence interval which did not contain zero (see Table 5). Lower reported levels of acknowledgement from healthcare professionals were associated with higher intuitive grief scores.

**Table 5:** Multiple stepwise linear regression for intuitive grief (*n* = 210)

	*B*	95% CI	SE *B*	*β*	*p*
**Variables**					
***Ultrasound viewing***					
Ultrasound viewed during pregnancy	1.5	(−0.01–3.03)	.77	.15	.05
Ultrasound not viewed during pregnancy	Ref	–	–	–	–
***Ethnicity***					
Other	1.17	(−0.12–2.35)	.60	−.13	.05
Australian	Ref	–	–	–	–
PAAS Quality of Attachment subscale	.10	(−0.02–0.22)	.06	.12	.09
Age at loss (in years)	.07	(−0.02–0.16)	.05	.07	.10
Number of surviving children at loss	−.20	(−0.67–0.27)	.24	−.05	.40
Agreement to the statement: “My role following the loss was to support my partner and family”	−.37	(−0.90–0.15)	.27	−.08	.16
CSS total score	.06	(0.4 – −0.01)	.04	.15	.10
Extent of acknowledgement for grief from family	.26	(−0.24–0.76)	.26	.11	.31
CMNI Self-Reliance subscale	−.14	(−0.29–0.02)	.08	−.12	.08
MRNI Toughness subscale	−.05	(−0.17–0.06)	.06	−.07	.37
***Workplace leave***					
Employment leave offered	4.98	(−1.2–11.16)	3.16	.73	.11
Employment leave not offered	4.93	(−1.3–11.19)	3.19	.62	.12
Did not inform employer of loss	Ref	–	–	–	–
***Other workplace supports***					
Other supports offered	−3.38	(−9.72–2.95)	3.23	−.57	.29
Other supports not offered	−3.78	(−10.19–2.63)	3.27	−.67	.25
Did not inform employer of loss	Ref	–	–	–	–
Extent of acknowledgement from healthcare professionals	**−.46**	**(−0.84 – −0.08)**	.20	−.18	.02

#### Model 3: Instrumental grief (GPI)

Sixteen variables met the 0.5 significance cut-off for inclusion in the final model for instrumental grief; of which, eight had confidence intervals which did not contain zero (see Table 6). While increased quality of attachment was associated with a slight decrease in men’s grief scores, higher scores on time in attachment were associated with an increase in grief. Although the supporter role itself was not associated with instrumental grief, men who perceived their supporter role as interfering more with their ability to grieve experienced higher levels of instrumental grief. Lower grief scores were associated with higher levels of total support. More specifically, higher perceived acknowledgement of men’s grief from their partner was associated with a reduction in grief. Higher endorsement of self-reliance masculine ideals was associated with higher levels of instrumental grief. Finally, men who did not inform their workplace of their loss had higher levels of grief in comparison to those who did; this was regardless of whether workplace leave was offered to those who informed their employer. However, the confidence intervals for these workplace factors were wide, indicating a degree of caution should be exercised regarding the strength of these relationships.

**Table 6:** Multiple stepwise linear regression for instrumental grief (*n* = 210)

	*B*	95% CI	SE *B*	*β*	*p*
**Variables**					
***Loss focus***					
Ectopic pregnancy	−.91	(−3.8–1.99)	1.48	−.03	.54
TOPFA	−.38	(−1.86–1.10)	.76	−.04	.62
Miscarriage	−1.12	(−2.43–0.20)	.67	−.12	.09
Stillbirth	.21	(−0.98–1.41)	.61	.02	.73
Neonatal death	Ref	–	–	–	–
***Ethnicity***					
Other	−.41	(−1.59–0.77)	.60	.06	.49
Australian	Ref	–	–	–	–
PAAS Quality of Attachment subscale	**−.15**	**(−0.30 – −0.01)**	.07	−.15	.03
PAAS Time in Attachment subscale	**.16**	**(0.04–0.27)**	.06	.17	<.01
Age at loss (in years)	−.84	(−0.17–0.01)	.05	−.12	.06
Importance of religion	−.25	(−0.57–0.08)	.17	−.11	.13
Marital satisfaction at the time of loss	.43	(−0.40–1.26)	.42	.07	.31
Agreement to the statement: “My role following the loss was to support my partner and family”	.49	(−0.05–1.03)	.27	.09	.07
Agreement to the statement: “I was unable to grieve, because I was too busy supporting everyone else”	**.51**	**(0.13–0.89)**	.20	.18	<.01
CSS total score	**−.08**	**(− 0.16 – − 0.01)**	.04	−.18	.03
Acknowledgement of grief from partner	**−.53**	**(−0.99 – − 0.06)**	.24	−.17	.03
Acknowledgement of grief from friends	.27	(−0.26–0.77)	.24	.07	.29
CMNI Self-Reliance subscale	**.19**	**(0.03–0.34)**	.08	.16	.02
MRNI Toughness subscale	.07	(−0.04–0.19)	.06	.08	.22
***Workplace leave***					.09
Employment leave offered	**−2.01**	**(−3.63 – −0.39)**	.83	−.14	.02
Employment leave not offered	**−2.25**	**(−3.98 – −0.52)**	.89	−.15	.01
Did not inform employer of loss	Ref	–	–	–	–
***Perceived degree of inclusion in the hospital***					.33
High level of inclusion	−.103	(−1.39–1.18)	.66	−.04	.88
Low level of inclusion	−.48	(−1.82–0.85)	.68	−.11	.48
No contact with a hospital	Ref	–	–	–	–

## Discussion

### Main findings and implications

This study, using multivariable linear regression analyses, explored relationships between men’s grief following pregnancy loss/neonatal death and a range of previously identified socioecological factors [36]. In relation to the severity of men’s grief (as measured by the PGS), men who had experienced previous losses, lower levels of social support and more time bonding with their baby during pregnancy had higher grief scores. Men who had lower marital satisfaction, little acknowledgement of their grief from friends, felt as though their role as a ‘supporter’ prevented them from grieving and experienced higher levels of acknowledgement from family also had higher grief scores; however, the precision of the strength of relationships for these factors was less certain. Men’s grief scores also differed depending on the type of loss experienced; however, again, the extent to which loss type impacted grief scores was also less certain.

Factors associated with men’s grief also differed depending on grief style. There was a high level of confidence that increased perceived support from healthcare professionals was associated with lower levels of intuitive grief. Results also indicated that viewing an ultrasound image of their baby during pregnancy, identifying with an ethnicity other than Australian, developing a higher quality of attachment to the baby during pregnancy, higher levels of overall social support, and lower endorsement of self-reliance could be relevant for intuitive grief. However, given the confidence intervals for these factors just crossed zero, further research is needed to confirm the direction of the associations. In relation to instrumental grief scores, men who had higher levels of social support, high quality of attachment to their baby during pregnancy, and acknowledgement of grief from their partner, had reduced instrumental grief. In contrast, perceptions of their supporter role interfering with their grief, higher tendencies toward self-reliance, as well as an increased amount of time spent bonding with their baby during pregnancy, were associated with higher levels of instrumental grief. Men who did not inform their workplace of their loss also had higher levels of instrumental grief than men who did, however the precision of these estimates was less certain. While it is possible that informing an employer leads to lower grief levels (e.g., through enhancing recognition of grief), this finding may also be reflective of the instrumental grief style itself, which typically involves coping in isolation and privacy [27].

These findings relating to grief styles imply that strategies to best support men may need to vary depending on men’s grieving style. For example, intuitive grievers may benefit from higher levels of healthcare professional support and acknowledgement in the hospital, whereas instrumental grievers may benefit more from external social supports and higher levels of partner acknowledgement for their grief. This idea is in line with research on grief styles, which suggests that intuitive grievers more frequently access professional counselling services, whereas instrumental grievers rely on informal social supports [27, 86]. However, this is not to say that counselling is unsuitable for instrumental grievers. Rather, traditional counselling services may need to better target and support the unique needs of instrumental grievers and use tailored marketing strategies to increase their appeal/accessibility among men [86–88]. In addition, receiving adequate informal social supports may be a useful first step to providing recognition and validation to instrumental grievers, which could then lead to accessing more formal support services where required.

Although men who had experienced an early gestation loss (before 20 weeks) had the lowest average grief score, their scores still met the cut-off for a high degree of grief. Standard deviations also indicated a wide variation in scores across loss types, supporting the view than grief is a highly individualised experience, not necessarily dependent on the gestational age of the baby [10, 12, 36]. Overall, men who experienced later-gestation loss (including stillbirth and TOPFA after 20 weeks’ gestation) had the highest average grief scores. Such high levels of grief may be related to both the unexpected nature of stillbirth, specific challenges associated with TOPFA, and with the stigma and disenfranchisement that many bereaved parents experience [1, 5–8, 10, 17, 89, 90]. In comparison to a neonatal death, which may be due to known medical complications and managed through a Neonatal Intensive Care Unit (NICU), parents who experience stillbirth continue to report variation in care received and availability of support services [42, 91–94].

Men’s role as a ‘supporter’ to their female partner has been a consistent finding across studies [10, 12, 13, 36, 39, 40, 60]. However, our findings suggest that this role in and of itself was not a substantial contributor to men’s grief intensity. Instead, it was the extent to which men perceived the supporter role to interfere with their grieving that was significant, particularly for instrumental grievers. Assuming a supporter role is not necessarily a negative contributor to the grief experience but, where this role takes precedence over men’s needs, it may become detrimental to their grief. It is therefore imperative that healthcare professionals are equipped to assist men to balance their desire and need to support their partner, while also addressing their grief and need for support. Healthcare professionals may assist men to achieve balance by not only providing them with tailored practical tips for supporting their partner but also acknowledging their grief and making efforts to provide active, ongoing support in the weeks/months following the loss.

In line with previous research, the degree of men’s attachment to their baby during pregnancy was associated with grief [10, 16, 36]. Although viewing an ultrasound was associated with instrumental grief, broader measures of attachment, including both time in attachment and quality of attachment, had stronger associations with grief in general. These findings are in contrast to early research suggesting that viewing an ultrasound and attending obstetric appointments were the main drivers of men’s attachment to a developing baby [24, 25, 37], demonstrating that many men develop a very early prenatal attachment to their baby.

Although the precision of the estimate was uncertain, one of the more unexpected findings was that higher levels of grief were associated with more acknowledgement from family. This relationship could be purely correlational, in that men who experienced higher grief sought more acknowledgement and support from family members. However, it could also be that although men received support from their family, the type of support received did not address their needs. For example, previous research suggests that although family members may be available to support men, the support may not be effective. Challenges to providing effective support reported by men have included a lack of understanding or unhelpful comments despite well-meaning intentions [10–12, 32, 95], feeling as though they needed to support their family members through their grief [10, 13, 16, 32, 95, 96], not feeling comfortable discussing their feelings with family members (where family referred to people other than their female partner) [12], and a desire for practical support (e.g., cooking, cleaning, childcare) as well as emotional support [16]. In line with research exploring the impact of pregnancy loss and neonatal death on extended family members including siblings and grandparents [97–101], this finding supports a family-centred approach to providing information and support for loss and grief, so that all family members involved in the experience of loss are better able to support one another.

### Strengths, limitations and future research

Previous research involving bereaved parents has noted difficulties in representing men’s perspectives, with female participants more often than not outweighing men [7, 9, 93, 101]. This study is one of the largest samples of men to have been surveyed on their experiences of grief following pregnancy loss and neonatal death in Australia. In line with father-inclusive practice recommendations [88, 102], targeting the research directly for ‘men/fathers’ specifically, rather than ‘parents’ collectively, was a successful approach. However, although the sample is sizeable, the convenience nature of sampling is open to potential bias in that participants may have been unique from other men who chose not to participate. For example, one third of participants in this study had experienced four or more previous losses. Participants were also recruited through advertisements disseminated by Australian pregnancy loss and neonatal death support organisations. This recruitment approach could constitute a sampling bias in that men who were not connected to these organisations would not have had access to the information to participate.

High levels of internal consistency were observed for the majority of included measures. However, a low Cronbach’s alpha was observed for the *Toughness* subscale of the MRNI. Although this measure of ‘toughness’ did not emerge as a significant predictor in any of the models in the current study, it may still be an important factor to consider, as a low alpha value may indicate that this measure did not adequately capture men’s experiences of needing to be ‘strong’ or ‘tough’ as reported in previous qualitative studies [10, 12, 13, 31, 36]. Future research could explore alternative ways to measure this construct and assess whether it is important in explaining men’s grief.

Although the majority of men reflected on losses within the last five years, this study relied on retrospective accounts of grief which may be open to recall bias, especially for the small number of losses which had occurred up to 20 years ago. Although we found no substantial differences in grief scores according to time since loss in our sample, changes in individual, community and health system/policy level support over time are likely to shape men’s grief outcomes. For example, the Australian government recently announced policy changes to allow parents up to 12 months of unpaid leave following a stillbirth [103]. This change is a substantial step forward for recognition of parents’ grief after stillbirth, and may ease the burden of grief on men. However, this research was conducted before these changes and future research is recommended to monitor trends in uptake and impact upon grief. Longitudinal studies which follow men during pregnancy and in the event of a pregnancy loss or neonatal death would be useful to identify the factors associated with grief at the time of loss, as well as to trial support services which may be useful.

The cultural diversity of the sample was also limited. Although men who identified as Australian had slightly higher levels of intuitive grief, no other associations were identified in relation to ethnicity. There is an ongoing and pressing need to examine the experiences of culturally and linguistically diverse men following pregnancy loss and neonatal death, as well as men in some countries where pregnancy and childbirth are still very much considered ‘women’s business’ [33, 60, 104]. This is despite increasing evidence of the health benefits for both mother and baby when male partners are engaged in pregnancy and birth [104–108]. Finally, although this study was open to non-heterosexual men, only one participant identified as bisexual, and none as gay or transgender. Given research to suggest that gay and transgender men may face unique challenges concerning pregnancy, birth and loss [109–112], there is a need for research specifically targeting the experiences of these under-represented groups.

## Conclusions

As this is one of the first studies to comprehensively explore multiple socioecological factors associated with men’s grief following pregnancy loss and neonatal death, many of the findings are relatively novel and require further research to understand the causal pathways underlying relationships. However, what is clear is that men often experience significant grief following a pregnancy loss or neonatal death. There is a need to initiate and trial support interventions targeted specifically to men and designed with consideration for the factors associated with men’s grief. Intervention strategies should engage individually with men both immediately in hospitals, and in the weeks/months following a loss, to ensure they have access to tailored support and services where these are needed. Intervention, particularly for intuitive grievers, could include formal brief assessment of men’s grief and mental health in the hospital and in the weeks/months following discharge (e.g., the Edinburgh Postnatal Depression Scale). Intervention, particularly for instrumental grievers, could also involve providing a follow-up telephone service specifically to men post-discharge from the hospital including referral to community-based supports where required, or delivering couples-based psychoeducation sessions to foster positive communication, mutual understanding of individual grief styles and information on supporting one another. At the service level, an intervention could include delivering father-inclusive training to healthcare professionals who work with couples experiencing pregnancy loss and neonatal death. To best assist men, genuine acknowledgement and engagement of men as equal partners throughout pregnancy, and in loss and grief, is required. Taking a public health or socioecological approach to understanding grief will also be beneficial in identifying target areas for strategies in all areas of men’s lives that may be affected by their grief.

## Supplementary Information

**Additional file 1.** Copy of the online survey.

## Data Availability

The datasets generated and analysed during the current study are not publicly available as ethical consent was not obtained from participants to do so, and the data cannot be anonymised sufficiently for public sharing. The data may be available from the corresponding author on reasonable request.

## Abbreviations

CMNI: Conformity to Masculine Norms Inventory
CSS: Crisis Support Scale
GPI: Grief Patterns Inventory
MRNI: Male Role Norms Inventory
NICU: Neonatal Intensive Care Unit
PAAS: Paternal Antenatal Attachment Scale
PGS: Perinatal Grief Scale
SD: Standard deviation
TOPFA: Termination of pregnancy for foetal anomaly
