# Supplementary material for: Factors contributing to men’s grief following pregnancy loss and neonatal death: further development of an emerging model in an Australian sample
Source: BMC Pregnancy Childbirth. 2021 Jan 7;21:29. doi: 10.1186/s12884-020-03514-6 (PMC7792062; doi:10.1186/s12884-020-03514-6)
Supplement: Supplementary file 1 — Additional file 1. Copy of the online survey. [file 12884_2020_3514_MOESM1_ESM.pdf]

Appendix 1. Copy of the online survey

THE UNIVERSITY of ADELAIDE

Australian men's grief following pregnancy loss and neonatal loss

Thank you for your interest in contributing to this important area of research.

What is the study about?

We want to improve understandings of men's grief following pregnancy loss or neonatal loss (the death of a baby within 28 days of birth) in Australia.

Many of the guidelines for supporting parents after infant loss are based largely on women's experiences, with little information on men's grief and support needs. This study may help to inform future guidelines in Australia, so fathers can be better supported in future.

Who is undertaking the study?

This project is being conducted by Kate Obst, forming part of the degree of a PhD/Master of Psychology (Health) at the University of Adelaide under the supervision of Dr Clemence Due, Dr Melissa Oxlad and A/Prof Philippa Middleton. Ms Obst is supported by an Australian Government Research Training Program Scholarship and a Westpac Scholars Trust 2018 Future Leaders Scholarship.

You can complete this survey if:

- You are an adult male (over 18 years of age)
- You have experienced the loss of a baby in Australia in the last 20 years

The loss of a baby includes:

- Ectopic pregnancy
- Miscarriage (before 20 weeks of pregnancy)
- Stillbirth (at or after 20 weeks of pregnancy, or over 400g in weight)
- The loss of an infant within the first 28 days of life (neonatal death)
- Termination/interruption of pregnancy due to life-threatening causes

What will I be asked to do, and how long will it take?

This survey will ask questions relating to your experience of grief following pregnancy/neonatal loss. It should take no longer than 30 minutes to complete. We will also ask if you are interested in being contacted for a follow-up interview. However, this is entirely voluntary and you are under no obligation to do so. Please note that there are no immediate benefits to participating.

Can I withdraw from the study?

Participation is completely voluntary. Even if you begin the survey, you can withdraw at any time by exiting the browser. However, once you have submitted your responses, you will not be able to withdraw them.

What will happen to my information?

Your responses to this survey are anonymous. However, if you provide your contact details for a potential follow-up interview, this information will be kept with your data, so we can build on your responses.

The results may be written up for publication to research journals, presentations at national or international conferences, and a short report for national pregnancy loss support organisations or participants who are interested. If you would like a copy of the results, you can provide your email address at the end of this survey, or via the research website: https://health.adelaide.edu.au/mens-grief

If you do provide any identifying information, this will not be included in any publications. The survey data will be stored securely for a period of five years at the University of Adelaide. Only the researchers named here will have access to this information.

Who do I contact if I have questions?

The researchers' contact details are as follows:

Kate Obst kate.obst@adelaide.edu.au or (08) 8313 6972
Dr Clemence Due clemence.due@adelaide.edu.au or (08) 8313 6069
Dr Melissa Oxlad melissa.oxlad@adelaide.edu.au
A/Prof Philippa Middleton philippa.middleton@adelaide.edu.au

What if I have a complaint or concerns?

Appendix 1. Copy of the online survey

This study has been approved by the Human Research Ethics Committee at the University of Adelaide (approval number H-2018-273).

If you have questions or problems with your participation, or wish to raise a complaint or concern, you should contact the Principal Investigator (Dr Clemence Due).

If you wish to speak with an independent person regarding concerns or a complaint, the University's policy on research involving human participants, or your rights as a participant, contact the Human Research Ethics Committee at:

Phone: (08) 8313 6028
Email: hrec@adelaide.edu.au
Post: Level 4, Rundle Mall Plaza, 50 Rundle Mall, ADELAIDE SA 5000

Any complaint or concern will be treated in confidence and fully investigated. You will be informed of the outcome.

If you would like to participate, please click 'next' below to begin. The submission of your responses will be taken as indication of your consent to participate.

Next

THE UNIVERSITY of ADELAIDE

If any of the following questions cause you distress, or you feel as though you need to speak to someone, please contact either:

Lifeline, on 13 11 14 or
MensLine, on 1300 78 99 78

Please note: We recommend completing this survey in one sitting (no more than 30 minutes). However, if you do need to take a short break, you can leave your responses in an open tab and return to them later.

If you need a longer break (i.e., hours or days), please finish the page you are currently on, and select 'next' to save your responses and proceed to the next page. From here, you will be able to close your browser and return to the survey at a later time to complete it. However, to return to your current position in the survey, you must be using the same device and browser that you used previously.

Prev Next

Demographic Information

Please answer the following questions relating to general demographic information as best you can. If none of the options provided apply to you, please select 'other' and fill in an appropriate response.

* **What is your current age** (in years)?

* **What is the highest level of education you have completed?**

- Primary School
- High School
- TAFE/Trade
- Undergraduate Degree (Bachelor)
- Postgraduate Degree (Masters/PhD)

Other (please specify)

Appendix 1. Copy of the online survey

* **To which ethnic background do you most identify?**

- [ ] Australian
- [ ] Australian Aboriginal
- [ ] Torres Strait Islander
- [ ] Aboriginal and Torres Strait Islander
- [ ] New Zealander
- [ ] Asian
- [ ] Indian
- [ ] Middle Eastern
- [ ] European
- [ ] North American
- [ ] South American
- [ ] Other (please specify)

* **Which of the following options best describes how you think of yourself?**

- ○ Straight (heterosexual)
- ○ Gay
- ○ Bisexual
- ○ Don't know
- ○ Rather not answer
- ○ Other (please specify)

* **Please select your current marital status**

- ○ In a relationship
- ○ Married
- ○ Divorced
- ○ Never married/single
- ○ Separated
- ○ Widowed

Other (please specify)

* **How important is religion/spirituality in your life?**

Not at all important	Not so important	Somewhat important	Very important	Extremely important
○	○	○	○	○

* **What is your postcode?**

* **What is your current employment status?**

- ○ Full time (35 hours or more per week)
- ○ Part time (less than 35 hours per week)
- ○ Casual
- ○ Student
- ○ Unemployed
- ○ Retired

Prev Next

Appendix 1. Copy of the online survey

* **What is your occupation?** (You may select more than one, if applicable)

- [ ] Manager
- [ ] Professional
- [ ] Technician or trade worker
- [ ] Community or personal services worker
- [ ] Sales worker
- [ ] Machine operator or driver
- [ ] Labourer
- [ ] Other (please specify)

Prev Next

Pregnancy and Loss History

Please keep in mind that 'pregnancy loss' refers to the loss of an unborn baby at any stage during pregnancy or labour. 'Neonatal loss' refers to the loss of a baby up to 28 days after a live birth. Multiple pregnancies are also included.

* **How many pregnancy losses or neonatal losses have you experienced?**

- ○ One
- ○ More than one

Prev Next

* **What type of pregnancy loss or neonatal loss have you experienced?**
If you are unsure, please make the best estimate that you can.

- ○ Ectopic pregnancy (defined as a pregnancy that implants outside the uterus/womb)
- ○ Termination/interruption of pregnancy due to life-threatening causes (at any stage of pregnancy)
- ○ Miscarriage (defined in Australia as a loss in-utero before 20 weeks of pregnancy)
- ○ Stillbirth (defined in Australia as a loss after 20 weeks of pregnancy, or over 400 grams in weight)
- ○ Neonatal loss (defined in Australia as a loss of an infant within 28 days after a live birth)

Prev Next

Appendix 1. Copy of the online survey

* **What types of pregnancy loss or neonatal loss have you experienced, and how many?**
Use the drop boxes to provide a number for the loss types that are relevant to you.
If you are unsure, please make the best estimate that you can.

	Number
**Ectopic pregnancy** (defined as a pregnancy that implants outside of the uterus/womb)	
**Termination/interruption of pregnancy for life-threatening causes** (at any stage of pregnancy)	
**Miscarriage** (defined in Australia as a loss in-utero before 20 weeks of pregnancy)	
**Stillbirth** (defined in Australia as a loss after 20 weeks of pregnancy, or over 400g in weight)	
**Neonatal loss** (defined as the loss of an infant up to 28 days after a live birth)	

Prev Next

Although you have experienced more than one pregnancy/neonatal loss, we ask that for the remainder of this survey you reflect only on the loss which has had the greatest impact on you.

* **Please select the type of loss you would like to reflect on for this survey:**

- Ectopic pregnancy (defined as a pregnancy that implants outside the uterus/womb)
- Termination/interruption of pregnancy for life-threatening causes (at any stage of pregnancy)
- Miscarriage (defined in Australia as a loss in-utero before 20 weeks of pregnancy)
- Stillbirth (defined in Australia as a loss after 20 weeks of pregnancy, or over 400 grams in weight)
- Neonatal loss (defined in Australia as a loss of an infant within 28 days after a live birth)

Prev Next

Stillbirth

Thank you. You have chosen to reflect on your experience of stillbirth. Please remember to answer all remaining questions with this loss in mind.

Prev Next

Appendix 1. Copy of the online survey

Stillbirth

At what stage of pregnancy (in closest weeks) did you lose your baby?
If you are unsure, please provide your best estimate, keeping in mind that a stillbirth occurs at or after 20 weeks, and the maximum length of a pregnancy is usually around 42 weeks.

How long ago did you lose your baby?
Please answer in the closest whole number of months or years (whichever is most applicable) since the loss.

Months

Years

How old were you (in years) at the time of your loss?

What was your marital status at the time of loss?

- ◯ In a relationship
- ◯ Married
- ◯ Divorced
- ◯ Never married/single
- ◯ Separated
- ◯ Other (please specify)

In general, how satisfied were you in your relationship with your partner around the time of your loss?

Not at all	A little bit	Moderately	Quite a bit	Very much	Not applicable
◯	◯	◯	◯	◯	◯

Did you attend obstetric or ultrasound appointments during the pregnancy that ended in stillbirth?

- ◯ Yes, always
- ◯ Yes, regularly
- ◯ Yes, occasionally
- ◯ No

At any point during the pregnancy that ended in stillbirth, did you view an ultrasound image/video of your baby?

- ◯ Yes
- ◯ No

Appendix 1. Copy of the online survey

How many surviving children did you have at the time of your loss?

- ◯ None
- ◯ One
- ◯ Two
- ◯ Three
- ◯ Four
- ◯ Five
- ◯ Six or more

Before the pregnancy that ended in stillbirth, had you used fertility treatments (either successfully or unsuccessfully) at any time in the past in attempt to fall pregnant?
E.g., intrauterine insemination (IUI), in vitro fertilisation (IVF); donor embryo, etc.

- ◯ Yes
- ◯ No

* **Was the pregnancy that ended in stillbirth planned?**

- ◯ Yes
- ◯ No

Prev Next

When trying for the pregnancy that ended in stillbirth, did it take longer than 12 months to fall pregnant?

- ◯ Yes
- ◯ No
- ◯ Unsure

Was the pregnancy that ended in stillbirth conceived using fertility treatments?
E.g., intrauterine insemination (IUI), in vitro fertilisation (IVF); donor embryo, etc.

- ◯ Yes
- ◯ No

Was the pregnancy that ended in stillbirth conceived using surrogacy arrangements?

- ◯ Yes
- ◯ No

Prev Next

Appendix 1. Copy of the online survey

These questions relate to your thoughts and feelings about the developing baby.

Thinking about the pregnancy that ended in loss, please tick one box only in answer to each question.

Please note: some of these questions may seem repetitive, however we ask that you please answer all of them to give us the best estimate of your typical behaviours during the pregnancy that resulted in a loss.

During the pregnancy, I thought about, or was preoccupied with the developing baby:

almost all the time	very frequently	frequently	occasionally	not at all

During the pregnancy, when I spoke about, or thought about the developing baby, I got emotional feelings which were:

very weak or non-existent	fairly weak	in between strong and weak	fairly strong	very strong

During the pregnancy, my feelings about the developing baby were:

very positive	mainly positive	mixed positive and negative	mainly negative	very negative

During the pregnancy, I had the desire to read about or get information about the developing baby. This desire was:

very weak or non-existent	fairly weak	in between strong and weak	fairly strong	very strong

During the pregnancy, I tried to picture in my mind what the developing baby actually looked like in the womb:

almost all the time	very frequently	frequently	occasionally	not at all

During the pregnancy, I thought of the developing baby mostly as:

a real little person with special characteristics	a baby like any other baby	a human being	a living thing	a thing not yet really alive

During the pregnancy, when I thought about the developing baby, my thoughts:

were always tender and loving	were mostly tender and loving	were a mixture of both tenderness and irritation	contained a fair bit of irritation	contained a lot of irritation

During the pregnancy, my ideas about possible names for the baby were:

very clear	fairly clear	fairly vague	very vague	I had no idea at all

During the pregnancy, when I thought about the developing baby, I got feelings which were:

very sad	moderately sad	a mixture of happiness and sadness	moderately happy	very happy

During the pregnancy, I thought about what kind of child the baby will grow into:

not at all	occasionally	frequently	very frequently	almost all the time

During the pregnancy, I felt:

very emotionally distant from the baby	moderately emotionally distant from the baby	not particularly emotionally close to the baby	moderately close emotionally to the baby	very close emotionally to the baby

Appendix 1. Copy of the online survey

When I imagined first seeing the baby after the birth, I expected that I would feel:

intense affection	mostly affection	affection, but I expect there may be a few aspects of the baby I will dislike	I expected there may be quite a few aspects of the baby I will dislike	I expected I might feel mostly dislike
○	○	○	○	○

After birth, I [would have] wanted to hold the baby:

immediately	after s/he had been wrapped in a blanket	after s/he has been washed	after a few hours, for things to settle down	the next day
○	○	○	○	○

During the pregnancy, I had dreams about the pregnancy or baby:

not at all	occasionally	frequently	very frequently	almost every night
○	○	○	○	○

During the pregnancy, I found myself feeling, or rubbing with my hand, the outside of my partner's stomach where the baby was:

a lot of times each day	at least once per day	occasionally	once only	not at all	N/A
○	○	○	○	○	○

Prev Next

Reactions to Your Loss

Each of the items below is a statement of thoughts and feelings which some people have concerning a loss such as yours.

There are no right or wrong responses to these statements.

For each item, select the number which best indicates the extent to which you agree or disagree with it. If you are not certain, use the "neither" category. However, please try to use this category only when you truly have no opinion.

If it has been some time since your loss, please think back to your reactions soon after your loss.

Please note: If you are unsure as to what the question is asking, please answer to the best of your ability, relating to your reactions most of the time.

I feel/felt depressed

Strongly agree	Agree	Neither agree nor disagree	Disagree	Strongly disagree
○	○	○	○	○

I find/found it hard to get along with certain people

Strongly agree	Agree	Neither agree nor disagree	Disagree	Strongly disagree
○	○	○	○	○

Appendix 1. Copy of the online survey

I feel/felt empty inside

Strongly agree	Strongly disagree	Neither agree nor disagree	Disagree	Strongly disagree
○	○	○	○	○

I can't/couldn't keep up with my normal activities

Strongly agree	Agree	Neither agree nor disagree	Disagree	Strongly disagree
○	○	○	○	○

I feel/felt a need to talk about the baby

Strongly agree	Agree	Neither agree nor disagree	Disagree	Strongly disagree
○	○	○	○	○

I am/was grieving for the baby

Strongly agree	Agree	Neither agree nor disagree	Disagree	Strongly disagree
○	○	○	○	○

I am/was frightened

Strongly agree	Agree	Neither agree nor disagree	Disagree	Strongly disagree
○	○	○	○	○

I have considered suicide since the loss

Strongly agree	Agree	Neither agree nor disagree	Disagree	Strongly disagree
○	○	○	○	○

I take/took medicine for my nerves

Strongly agree	Agree	Neither agree nor disagree	Disagree	Strongly disagree
○	○	○	○	○

I very much miss/missed the baby

Strongly agree	Agree	Neither agree nor disagree	Disagree	Strongly disagree
○	○	○	○	○

I feel/felt I have adjusted well to the loss

Strongly agree	Agree	Neither agree nor disagree	Disagree	Strongly disagree
○	○	○	○	○

It is/was painful to recall memories of the loss

Strongly agree	Agree	Neither agree nor disagree	Disagree	Strongly disagree
○	○	○	○	○

I get/got upset when I think about the baby

Strongly agree	Agree	Neither agree nor disagree	Disagree	Strongly disagree
○	○	○	○	○

I cry/cried when I think about him/her

Strongly agree	Agree	Neither agree nor disagree	Disagree	Strongly disagree
○	○	○	○	○

I feel/felt guilty when I think about the baby

Strongly agree	Agree	Neither agree nor disagree	Disagree	Strongly disagree
○	○	○	○	○

I feel/felt physically ill when I think about the baby

Strongly agree	Agree	Neither agree nor disagree	Disagree	Strongly disagree
○	○	○	○	○

I feel/felt unprotected in a dangerous world since he/she died

Strongly agree	Agree	Neither agree nor disagree	Disagree	Strongly disagree
○	○	○	○	○

Appendix 1. Copy of the online survey

I try/tried to laugh, but nothing seems/seemed funny anymore

Strongly agree	Agree	Neither agree nor disagree	Disagree	Strongly disagree
○	○	○	○	○

Time passes/passed slowly since the baby died

Strongly agree	Agree	Neither agree nor disagree	Disagree	Strongly disagree
○	○	○	○	○

The best part of me died with the baby

Strongly agree	Agree	Neither agree nor disagree	Disagree	Strongly disagree
○	○	○	○	○

I have let people down since the baby died

Strongly agree	Agree	Neither agree nor disagree	Disagree	Strongly disagree
○	○	○	○	○

I feel/felt worthless since he/she died

Strongly agree	Agree	Neither agree nor disagree	Disagree	Strongly disagree
○	○	○	○	○

I blame/blamed myself for the baby's death

Strongly agree	Agree	Neither agree nor disagree	Disagree	Strongly disagree
○	○	○	○	○

I get/got cross at my friends and relatives more than I should

Strongly agree	Agree	Neither agree nor disagree	Disagree	Strongly disagree
○	○	○	○	○

Sometimes I feel/felt like I need a professional counsellor to help me get my life back together again

Strongly agree	Agree	Neither agree nor disagree	Disagree	Strongly disagree
○	○	○	○	○

I feel/felt as though I'm just existing and not really living since he/she died

Strongly agree	Agree	Neither agree nor disagree	Disagree	Strongly disagree
○	○	○	○	○

I feel/felt so lonely since he/she died

Strongly agree	Agree	Neither agree nor disagree	Disagree	Strongly disagree
○	○	○	○	○

I feel/felt somewhat apart and remote, even among friends

Strongly agree	Agree	Neither agree nor disagree	Disagree	Strongly disagree
○	○	○	○	○

It's safer not to love

Strongly agree	Agree	Neither agree nor disagree	Disagree	Strongly disagree
○	○	○	○	○

I find/found it difficult to make decisions since the baby died

Strongly agree	Agree	Neither agree nor disagree	Disagree	Strongly disagree
○	○	○	○	○

I worry/worried about what my future will be like

Strongly agree	Agree	Neither agree nor disagree	Disagree	Strongly disagree
○	○	○	○	○

Appendix 1. Copy of the online survey

Being a bereaved parent means being a "Second-Class Citizen"

Strongly agree	Agree	Neither agree nor disagree	Disagree	Strongly disagree

It feels/felt great to be alive

Strongly agree	Agree	Neither agree nor disagree	Disagree	Strongly disagree

Prev Next

Reactions to Your Loss (continued)

Please rate each of the following statements based on how you responded after your loss **most** of the time.

If it has been some time since your loss, please think back to your reactions soon after your loss.

Please note: If you are unsure as to what the question is asking, please answer to the best of your ability, relating to your reactions most of the time.

I am/was more emotional than most people I know

Strongly disagree	Disagree	Neither agree nor disagree	Agree	Strongly agree

It seems/seemed natural for me to cry and show my feelings to others

Strongly disagree	Disagree	Neither agree nor disagree	Agree	Strongly disagree

It helps/helped me to express my grief through tears

Strongly disagree	Disagree	Neither agree nor disagree	Agree	Strongly agree

Although I know that I am/was grieving in my own way, others may think that I am cold and unfeeling

Strongly disagree	Disagree	Neither agree nor disagree	Agree	Strongly agree

I don't/didn't seem to feel things as deeply as most other people I know

Strongly disagree	Disagree	Neither agree nor disagree	Agree	Strongly agree

I appreciate/d it when others encourage me to share my innermost feelings about my loss with them

Strongly disagree	Disagree	Neither agree nor disagree	Agree	Strongly agree

I have been/was told that I am avoiding my grief even though I don't think that I am

Strongly disagree	Disagree	Neither agree nor disagree	Agree	Strongly agree

Appendix 1. Copy of the online survey

Even though I have/had returned to my normal routine, I continue to be overwhelmed by strong and painful feelings

Strongly disagree	Disagree	Neither agree nor disagree	Agree	Strongly agree
○	○	○	○	○

I believe that a bereavement support group is (would be) very helpful to me

Strongly disagree	Disagree	Neither agree nor disagree	Agree	Strongly agree
○	○	○	○	○

I resent/ed efforts to get me to show feelings that I really don't have

Strongly disagree	Disagree	Neither agree nor disagree	Agree	Strongly agree
○	○	○	○	○

I would rather talk about "issues" related to my loss than feelings about my loss

Strongly disagree	Disagree	Neither agree nor disagree	Agree	Strongly agree
○	○	○	○	○

I would describe myself as more intellectual than emotional

Strongly disagree	Disagree	Neither agree nor disagree	Agree	Strongly agree
○	○	○	○	○

I don't/didn't like others knowing how upset I am by my loss

Strongly disagree	Disagree	Neither agree nor disagree	Agree	Strongly agree
○	○	○	○	○

I often disguise/d how I'm really feeling inside

Strongly disagree	Disagree	Neither agree nor disagree	Agree	Strongly agree
○	○	○	○	○

Prev Next

* **Were you employed at the time of your loss?**

- ○ Yes
- ○ No

Prev Next

* **How soon did you return to work (in any capacity) following your loss?**

- ○ The next day
- ○ Within a couple of days
- ○ Within a week
- ○ Within two weeks
- ○ Within one month
- ○ Within 2-3 months
- ○ Within 6 or more months
- ○ I did not return to work
- ○ I have not yet returned to work, but plan to soon

Prev Next

Appendix 1. Copy of the online survey

Can you tell us why you did not return to work? (Optional)
If you prefer not to answer this question, please select "next" below.

Prev Next

* **Did you inform your workplace of your loss?**

- Yes
- No

Prev Next

* **Were you offered any employment leave by your workplace following your loss?**

- Yes
- No

Prev Next

What type of employment leave were you offered?

- Sick/carers leave
- Compassionate/bereavement leave
- Parental leave
- Annual leave
- Leave without pay
- Other (please specify)

Did you use the leave or support offered to you?

- Yes
- No

Prev Next

Did your workplace offer you any other types of support following after you loss?
For example, counselling/referral to a counsellor, flexibility in work hours, etc.?

- Yes
- No

If you answered yes to the above, please expand.
If you answered no, please leave blank and move to the next question.

In your opinion, could your workplace have offered you anything else to make your transition back to work easier?

Prev Next

Appendix 1. Copy of the online survey

The Hospital Experience

* **Did you have contact with a hospital as part of your loss experience?**

○ Yes

○ No

Prev | Next

To what extent did you feel as though healthcare professionals (e.g., doctors, nurses, obstetricians, midwives, social workers, chaplains) included you as part of the pregnancy/neonatal loss experience at the hospital?

Not at all	A little bit	Moderately	Quite a bit	Extremely
○	○	○	○	○

In the hospital, were you spoken to about, or offered any information on, men's grief, what to expect, and/or support options available to you following your loss?

○ Yes

○ No

What was, or would have been, most helpful for you at the hospital?

Do you have any further comments about your experiences at hospital?

Prev | Next

Support and Relationships

We are interested in the help that you received from **family and friends** following your loss. Please answer the questions that follow by selecting the most appropriate response.

Although you may have received different types of help from different family members and friends, please answer these questions in relation to the support that was available to you **most** of the time.

Whenever you wanted to talk, how often was there someone willing to listen following your loss?

Never	Very seldom	Seldom	Sometimes	Often	Very often	Always
○	○	○	○	○	○	○

Did you have personal contact with other bereaved parents following your loss?

Never	Very seldom	Seldom	Sometimes	Often	Very often	Always
○	○	○	○	○	○	○

Were you able to talk about your thoughts and feelings following your loss?

Never	Very seldom	Seldom	Sometimes	Often	Very often	Always
○	○	○	○	○	○	○

Appendix 1. Copy of the online survey

Were people sympathetic and supportive following your loss?

Never	Very seldom	Seldom	Sometimes	Often	Very often	Always
○	○	○	○	○	○	○

Were people helpful in a practical way following your loss?

Never	Very seldom	Seldom	Sometimes	Often	Very often	Always
○	○	○	○	○	○	○

Did people you expect to be supportive make you feel worse at any time following your loss?

Never	Very seldom	Seldom	Sometimes	Often	Very often	Always
○	○	○	○	○	○	○

Overall, were you satisfied with the support you received from family and friends following the loss?

Never	Very seldom	Seldom	Sometimes	Often	Very often	Always
○	○	○	○	○	○	○

Prev Next

Please rate the extent to which you agree with the following statement:

"My role following the loss was to support my partner and family"

Strongly disagree	Disagree	Neither agree nor disagree	Agree	Strongly agree
○	○	○	○	○

Please rate the extent to which you agree with the following statement:

"I was unable to grieve, because I was too busy supporting everyone else"

Strongly disagree	Disagree	Neither agree nor disagree	Agree	Strongly agree
○	○	○	○	○

To what extent did you feel your grief was acknowledged and recognised by your partner?

Not at all	A little bit	Moderately	Quite a bit	Extremely
○	○	○	○	○

To what extent did you feel your grief was acknowledged and recognised by your family members?

Not at all	A little bit	Moderately	Quite a bit	Extremely
○	○	○	○	○

To what extent did you feel your grief was acknowledged and recognised by your friends?

Not at all	A little bit	Moderately	Quite a bit	Extremely
○	○	○	○	○

To what extent did you feel your grief was acknowledged and recognised by the wider community?

Not at all	A little bit	Moderately	Quite a bit	Extremely
○	○	○	○	○

To what extent did you feel your grief was acknowledged and recognised by health professionals?
(e.g., doctors, nurses, obstetricians, midwives, social workers, hospital chaplains)

Not at all	A little bit	Moderately	Quite a bit	Extremely
○	○	○	○	○

Do you have any further comments about these questions?

Prev Next

Appendix 1. Copy of the online survey

* **If you have experienced more than one pregnancy loss or neonatal loss, would you like to comment further on these experiences?**

◯ Yes

◯ No

◯ Not applicable / I have only experienced one loss

Prev Next

Please comment on your other experience/s of loss, in terms of your grief, support, or anything else you feel is important.

Prev Next

Seeking Help

Thinking about your own actions, feelings and beliefs, please indicate how much you personally agree or disagree with each statement by the most appropriate response. There are no correct or wrong answers to the items. You should give the responses that most accurately describe your personal actions, feelings and beliefs. It is best if you respond with your first impression when answering.

Please note: the following questions do not relate directly to your experience of loss. Instead, please answer them in relation to your general actions and beliefs.

I never ask for help

Strongly disagree	Disagree	Agree	Strongly agree
◯	◯	◯	◯

It bothers me when I have to ask for help

Strongly disagree	Disagree	Agree	Strongly agree
◯	◯	◯	◯

I ask for help when I need it

Strongly disagree	Disagree	Agree	Strongly agree
◯	◯	◯	◯

I am not ashamed to ask for help

Strongly disagree	Disagree	Agree	Strongly agree
◯	◯	◯	◯

I hate asking for help

Strongly disagree	Disagree	Agree	Strongly agree
◯	◯	◯	◯

It's important for a man to take risks, even if he might get hurt

Strongly disagree	Disagree	More or less disagree	Undecided	More or less agree	Agree	Strongly agree
◯	◯	◯	◯	◯	◯	◯

When the going gets tough, men should get tough

Strongly disagree	Disagree	More or less disagree	Undecided	More or less agree	Agree	Strongly agree
◯	◯	◯	◯	◯	◯	◯

Appendix 1. Copy of the online survey

I think a young man should try to be physically tough, even if he's not big

Strongly disagree	Disagree	More or less disagree	Undecided	More or less agree	Agree	Strongly agree
○	○	○	○	○	○	○

Prev Next

Is there anything else you would like to add/say in regards to your experience following pregnancy/neonatal loss that you feel this survey has not covered? (optional)

Do you have any feedback on this survey? (optional)

Prev Next

Follow-Up Option

* **Are you interested in being contacted to potentially participate in a follow-up interview at a later date?**

○ Yes

○ No

Prev Next

So we can contact you about potentially participating in a follow-up interview, please provide an email address that you regularly check, and then select "next"

Prev Next

Thank you, this is the end of the survey. Your time is greatly appreciated.

If you would like to be provided with a copy of the results, please click here to provide your email.

If you would like to find out more about this program of research, please visit our research webpage at: **ENTER LINK**

If you feel as though you need to speak to someone, please contact Lifeline on 13 11 14, or MensLine on 1300 78 99 78

To submit your responses, please select "done" at the bottom of the page.

Prev Done
